# Vascular Tree Segmentation in Medical Images Using Hessian-Based Multiscale Filtering and Level Set Method

**DOI:** 10.1155/2013/502013

**Published:** 2013-11-19

**Authors:** Jiaoying Jin, Linjun Yang, Xuming Zhang, Mingyue Ding

**Affiliations:** Department of Biomedical Engineering, School of Life Science and Technology, Key Laboratory of Image Processing and Intelligent Control of Education Ministry of China, Huazhong University of Science and Technology, Wuhan 430074, China

## Abstract

Vascular segmentation plays an important role in medical image analysis. A novel technique for the automatic extraction of vascular trees from 2D medical images is presented, which combines Hessian-based multiscale filtering and a modified level set method. In the proposed algorithm, the morphological top-hat transformation is firstly adopted to attenuate background. Then Hessian-based multiscale filtering is used to enhance vascular structures by combining Hessian matrix with Gaussian convolution to tune the filtering response to the specific scales. Because Gaussian convolution tends to blur vessel boundaries, which makes scale selection inaccurate, an improved level set method is finally proposed to extract vascular structures by introducing an external constrained term related to the standard deviation of Gaussian function into the traditional level set. Our approach was tested on synthetic images with vascular-like structures and 2D slices extracted from real 3D abdomen magnetic resonance angiography (MRA) images along the coronal plane. The segmentation rates for synthetic images are above 95%. The results for MRA images demonstrate that the proposed method can extract most of the vascular structures successfully and accurately in visualization. Therefore, the proposed method is effective for the vascular tree extraction in medical images.

## 1. Introduction

 Accurate segmentation and quantification of vascular structures in medical images is a critical task for clinical practices such as computer-aided diagnosis, treatment, surgical planning, and navigation. However, it is highly challenging to extract vascular structures in 2D and 3D medical images. The reasons lie in two aspects. On one hand, some vascular structures involve numerous vascular branches and complex patterns [1]. On the other hand, noise, variations in intensities, and low image contrast pose difficulties in vascular tree extraction [2].

Various extraction techniques have been proposed for vascular tree segmentation, that is, pattern recognition techniques, model-based approaches, mathematical morphology, multiscale filtering approaches, vessel tracking, and matched filtering (see Kirbas and Quek [3] and Lesage et al. [4] for comprehensive reviews). Almost all the vascular extraction techniques take advantage of the characteristics of tubular-like or line-like structure of vessels. Among existing vascular extraction methods, Hessian-based multiscale filtering has received much attention [1, 5–12]. These methods share a common idea that the images are convolved with 2D or 3D Gaussian filters at multiple scales, and the eigenvalues of the Hessian matrix at each pixel or voxel are analyzed in terms of a response function to determine the shape of the local structures in the images [13]. The response of the Hessian-based multiscale filtering will be strongest when the scale of the filter matches the size of the local structures, which means scale selection is keeping with the strongest response among multiple scales. Thus, local structures can be extracted using the local strongest response. However, the Hessian-based multiscale filtering is only based on geometry structures of the vessels, which will lead to the discontinuous, even fake vascular structures [1]. Furthermore, Gaussian filter convolution with the image tends to blur vessel boundaries and thus makes the scale selection inaccurate. To address these problems, we use morphological top-hat transformation and Hessian-based multiscale filtering to enhance vascular structures in medical images and an improved level set method involving an external constrained term related to the standard deviation *σ* of Gaussian function to extract vascular structures from the enhanced images since the blur level of vessel boundaries is associated with *σ* [14].

The paper is structured in five sections; Section 2 describes morphological top-hat transformation and Hessian-based multiscale filtering for vessel enhancement. Vessel segmentation with the improved level set method is presented in Section 3.  Section 4 provides some experimental results for synthetic and clinical medical images, as well as evaluation of robustness and segmentation accuracy of the proposed method. The conclusions and future directions are given in Section 5.

## 2. Vessel Enhancement

 The presence of numerous nonvascular structures in clinical medical images such as liver and kidney, will negatively affect the extraction of vascular structures. Considering that morphological top-hat transformation is a powerful technique for image enhancement, especially in extracting bright features from a dark background [15–19], it is adopted in our method to suppress nonvascular structures by using a structuring element larger than the maximum vessel scale in the medical images.

Following the morphological top-hat transformation, the Hessian-based multiscale filtering [5] is used for enhancing the medical image. The filter is based on eigenvalue analysis of the scale space of the Hessian matrix. The eigenvalues and eigenvectors of Hessian matrix are closely related to vascular intensity and direction. For a 3D input image *I*, Hessian matrix is a 3 × 3 matrix composed of second-order partial derivatives of the input image *I*:

(1) $$\begin{array}{c} \nabla^{2}I=\left[\begin{array}{ccc} \frac{\partial^{2}I}{\partial x^{2}} & \frac{\partial^{2}I}{\partial x\partial y} & \frac{\partial^{2}I}{\partial x\partial z}\\ \frac{\partial^{2}I}{\partial y\partial x} & \frac{\partial^{2}I}{\partial y^{2}} & \frac{\partial^{2}I}{\partial y\partial z}\\ \frac{\partial^{2}I}{\partial z\partial x} & \frac{\partial^{2}I}{\partial z\partial y} & \frac{\partial^{2}I}{\partial z^{2}} \end{array}\right]. \end{array}$$

In practice, the second-order partial derivatives of input image *I* at a point (*x*, *y*, *z*) are defined as a convolution with derivatives of Gaussian filter at scale *σ*:

(2) $$\begin{array}{c} I\left(x,y,z,\sigma \right)=G\left(x,y,z,\sigma \right)*I\left(x,y,z\right),\\ G\left(x,y,z,\sigma \right)=\frac{1}{\sqrt{{\left(2\pi \sigma^{2}\right)}^{3}}}\exp\left(-\frac{x^{2}+y^{2}+z^{2}}{2\sigma^{2}}\right), \end{array}$$

where *G*(*x*, *y*, *z*, *σ*) denotes a Gaussian convolution kernel at scale *σ*. Let the eigenvalues of ∇^2^ *I* be *λ*_1_, *λ*_2_, and *λ*_3_ (|*λ*_1_| ≤ |*λ*_2_| ≤ |*λ*_3_|), and their corresponding eigenvectors be *e*_1_, *e*_2_, and *e*_3_, respectively.  Table 1 summarizes the relations between *λ*_*i*_ and orientations of different structures in the image.

Based on the eigenvalues of ∇^2^ *I*, the dissimilarity measures *R*_*B*_ and *R*_*A*_ are defined as

(3) $$\begin{array}{c} R_{B}=\frac{\left|\lambda_{1}\right|}{\sqrt{\left|\lambda_{2}\lambda_{3}\right|}},\\ R_{A}=\frac{\left|\lambda_{2}\right|}{\left|\lambda_{3}\right|}. \end{array}$$

The first ratio *R*_*B*_ accounts for the deviation for a blob-like structure, but it cannot distinguish between a line-like and a plate-like pattern. The second ratio *R*_*A*_ is applied for distinguishing between plate-like and line-like structures. In order to reduce the response of the background pixels, Frangi et al. used the Frobenius norm of the Hessian matrix to define the measure of “second-order structureness” [5] as follows:

(4) $$\begin{array}{c} S=\sqrt{\lambda_{1}^{2}+\lambda_{2}^{2}+\lambda_{3}^{2}}. \end{array}$$

Assuming a bright blood image, the vesselness function can be defined as follows:

(5) $$\begin{array}{c} \nu_{o}\left(\sigma \right)=\{\begin{array}{cc} 0,\text{if}\;\lambda_{2}>0\;\text{or}\;\lambda_{3}>0, & \\ \left(1-\exp\left(-\frac{R_{A}^{2}}{2\alpha^{2}}\right)\right)\exp\left(-\frac{R_{B}^{2}}{2\beta^{2}}\right) & \\ \;\times \left(1-\exp\left(-\frac{S^{2}}{2c^{2}}\right)\right),\;\text{otherwise}, & \end{array} \end{array}$$

where *α*, *β*, and *c* are thresholds which control the sensitivity of the filter to the measures *R*_*A*_, *R*_*B*_, and *S*. The filter is applied at multiple scales, and the maximum response is selected to be a final estimate of vesselness. Consider

(6) $$\begin{array}{c} \nu_{I}=\underset{\sigma_{\min}\leq \sigma \leq \sigma_{\max}}{\max}\left\{\nu_{o}\left(\sigma \right)\right\}, \end{array}$$

where *σ*_min⁡_ and *σ*_max⁡_ are the minimum and maximum scales at which relevant structures are expected to be found. The choice of the two values must ensure that they will cover the range of vessel widths [5]. The maximum response output *ν*_*I*_ is the enhanced image which corresponds to line-like structures.

For 2D images, we use the following vesselness measure defined by Frangi et al. [5] which follows from the same reasoning as used for 3D:

(7) $$\begin{array}{c} \nu_{o}\left(\sigma \right)=\{\begin{array}{cc} 0, & \text{if}\;\lambda_{2}>0,\\ \exp\left(-\frac{R_{B}^{2}}{2\beta^{2}}\right)\left(1-\exp\left(-\frac{S^{2}}{2c^{2}}\right)\right), & \text{otherwise}, \end{array} \end{array}$$

where *R*_*B*_ = *λ*_1_/*λ*_2_ is the blobness measure in 2D images.

## 3. Vessel Segmentation

### 3.1. Active Contour Methods

 Active contour methods have been popular in a wide range of problems including visual tracking and image segmentation since they were first proposed in 1988 by Kass et al. [20]. The basic idea of active contour methods is to evolve a curve from a given initial state towards an object boundary. In this paper, we used region-based active contour methods under the level set framework to segment vascular trees. Region-based active contour methods assume intensities of image regions to be constants. Compared with edge-based methods, region-based approaches have many advantages such as robustness and insensitivity to image noise [21].

In region-based active contour methods, a curve is iteratively evolved by optimizing an objective function to find the boundary *C* of an object. Let the bounded open subset *Ω* ⊂ *R* ^2^ represent the image domain. Each image is defined as *I* : *Ω* → *R*, and (*x*, *y*) ∈ *Ω* is a spatial variable representing a single point within the image domain *Ω*. In the level set framework, the boundary *C* is embedded in a higher-dimensional function. For example, a simple curve on a 2D plane can be embedded in a 3D surface *ϕ*. By convention, *C* is represented as the zero-level-set of *ϕ* such that the curve is located where *ϕ* crosses a plane at the 0th level. Thus, on the interior of *C*, *ϕ* < 0, and on the exterior of *C*, *ϕ* > 0. During the segmentation process, the function *ϕ*(*x*, *y*) is evolved rather than explicitly evolving the boundary itself when using a parametric boundary representation. The level set evolution equation is given by

(8) $$\begin{array}{c} \frac{\partial \phi \left(x,y\right)}{\partial t}+F\left|\nabla \phi \left(x,y\right)\right|=0, \end{array}$$

where *F* is speed function. In our implementation, *ϕ*(*x*, *y*) is initially represented as a signed distance function of the boundary and is evolved via the optimization of an objective function representing the goal of segmentation.

It is well known that level set methods are the most widely used way to represent a contour because of their simple implementation. In addition, it allows very complex curve behavior and automatic topology adaptation [22]. But the primary drawback of level set methods is that they are slow to compute. In this work, we borrow the idea of Lankton's work [22] to implement our approach with the sparse field method (SFM) proposed by Whitaker [23] which allows one to implement level set active contours efficiently. The objective function used in this work for vascular tree extraction will be described in Section 3.2.

### 3.2. Vascular Tree Segmentation

 In this paper, we use Chan-Vese model [24], a region-based active contour model, to overcome the drawback of Hessian-based multiscale filtering. The object of Chan-Vese model is to minimize the objective function *F*_cv_(*c*_1_, *c*_2_, *C*) defined by

(9) Fcv(c1,c2,C)=μ·Length(C)+α·Area(inside(C))+λ1∫inside(C)|u0(x,y)−c1|2dx dy+λ2∫outside(C)|u0(x,y)−c2|2dx dy,

where *c*_1_ and *c*_2_ denote the average intensity of pixels inside *C* and outside *C* (i.e., *c*_1_ = *c*_1_(*C*), *c*_2_ = *c*_2_(*C*)), respectively. The Heaviside function *H* and the Dirac Delta function *δ*_0_ will be used to partition the level set function. The objective function can be rewritten in terms of *ϕ*(*x*, *y*) as

(10) Fcv(c1,c2,ϕ) =μ∫Ωδ0(ϕ(x,y))dx dy+α∫ΩH(ϕ(x,y))dx dy  +λ1∫Ω|u0(x,y)−c1|2H(ϕ(x,y))dx dy  +λ2∫Ω|u0(x,y)−c2|2H(1−ϕ(x,y))dx dy.

Since Gaussian convolution tends to blur vessel boundaries and makes scale selection inaccurate, and the blur level of vessel boundaries is associated with standard deviation *σ* of Gaussian function, we define a new class of external constrained term *F*_*σ*_ related to *σ*. *F*_*σ*_ is the penalty on the evolution distance from the initial contour *C*_0_ which is obtained by using Otsu's thresholding [25] method on the enhanced image *ν*_*I*_. Here, *F*_*σ*_ is defined as

(11) $$\begin{array}{c} F_{\sigma }=B\left(x,y\right)\cdot F_{\text{cv}}\left(c_{1},c_{2},C\right),\\ B\left(x,y\right)=\{\begin{array}{cc} 0, & \text{if}\;\;\left|C_{t}\left(x,y\right)-C_{0}\right|<d_{\sigma },\\ -1, & \text{otherwise}, \end{array} \end{array}$$

where *σ* is the maximum standard deviation of Gaussian function used in Hessian-based multiscale filtering and *d*_*σ*_ is a preset value for the largest contour evolution distance (*d*_*σ*_ is set to *σ*_max⁡_/2, in our paper). |*C*_*t*_(*x*, *y*) − *C*_0_| is the evolution distance in a point (*x*, *y*) from the initial contour *C*_0_.

By combining *F*_cv_ with *F*_*σ*_, the new objective function is represented as

(12) Fnew=Fcv(c1,c2,C)+Fσ=Fcv(c1,c2,C)+B(x,y)·Fcv(c1,c2,C),

and it will be rewritten in terms of *ϕ*(*x*, *y*) as follows:

(13) $$\begin{array}{c} F_{\text{new}}=F_{\text{cv}}\left(c_{1},c_{2},\phi \right)+B\left(x,y\right)\cdot F_{\text{cv}}\left(c_{1},c_{2},\phi \right). \end{array}$$

The evolution follows that when evolution distance in point (*x*, *y*) |*C*_*t*_(*x*, *y*) − *C*_0_| < *d*_*σ*_, *B*(*x*, *y*) = 0, *F*_*σ*_ = 0, and *F*_new_ = *F*_cv_(*c*_1_, *c*_2_, *C*), where the contour *C*_*t*_ at point (*x*, *y*) can be evolved as the Chan-Vese model [24]. Meanwhile, when |*C*_*t*_(*x*, *y*) − *C*_0_| > *d*_*σ*_, *B*(*x*, *y*) = −1, *F*_*σ*_ = −*F*_cv_(*c*_1_, *c*_2_, *C*), and *F*_new_ = 0, where the contour *C*_*t*_ at point (*x*, *y*) will stop evolution. In other words, *d*_*σ*_ is the largest permissible evolution distance (i.e., *d*_*σ*_ = *σ*_max⁡_/2). Obviously, *B*(*x*, *y*) can control the contour evolution to avoid the segmentation leakage of nonvascular structures and blurred boundaries caused by Gaussian convolution.

If one regularizes the Heaviside function *H* and the Dirac Delta function *δ*_0_ by two suitable smooth functions *H*_*ε*_ and *δ*_*ε*_, the evolving equation will be obtained as

(14) ∂ϕ∂t=δε(ϕ)[μ∇·∇ϕ|∇ϕ|−α−λ1(u0−c1)2+λ2(u0−c2)2]+B(x,y)·δε(ϕ)×[μ∇·∇ϕ|∇ϕ|−α−λ1(u0−c1)2+λ2(u0−c2)2]

with the natural boundary condition [26]. In addition

(15) $$\begin{array}{c} \phi \left(x,y,0\right)=\phi_{0}\left(x,y\right),\;\frac{\delta \phi }{\left|\nabla \phi \right|}\frac{\partial \phi }{\partial \vec{n}}=0\;\mathrm{on}\;\;\partial \Omega ,\\ c_{1}\left(\phi \right)=\frac{\int_{\Omega }u_{0}\left(x,y\right)H\left(\phi \left(x,y\right)\right)dx\;dy}{\int_{\Omega }H\left(\phi \left(x,y\right)\right)dx\;dy},\\ c_{2}\left(\phi \right)=\frac{\int_{\Omega }u_{0}\left(x,y\right)H\left(1-\phi \left(x,y\right)\right)dx\;dy}{\int_{\Omega }H\left(1-\phi \left(x,y\right)\right)dx\;dy}. \end{array}$$

In general, *μ* ≥ 0, *ν* ≥ 0, *λ*_1_ > 0, and *λ*_2_ > 0 are fixed parameters. As suggested in [24], these parameters are set as *α* = 0, *λ*_1_ = *λ*_2_ = 1. Then the evolving equation can be simplified as

(16) ∂ϕ∂t=δε(ϕ)[μ∇·∇ϕ|∇ϕ|−(u0−c1)2+(u0−c2)2]+B(x,y)·δε(ϕ)×[μ∇·∇ϕ|∇ϕ|−(u0−c1)2+(u0−c2)2].

We define regularizations of *δ*_*ε*_ and *H*_*ε*_ (where *δ*_*ε*_(*x*) = (∂/∂*x*) *H*_*ε*_(*x*)) as

(17) $$\begin{array}{c} \delta_{\varepsilon }\left(x\right)=\{\begin{array}{cc} 1, & \text{if}\;\;x=0,\\ 0, & \text{if}\;\left|x\right|>\varepsilon ,\\ \frac{1}{\pi }\frac{\varepsilon }{\varepsilon^{2}+x^{2}}, & \text{if}\;\left|x\right|\leq \varepsilon , \end{array} \end{array}$$

(18) $$\begin{array}{c} H\left(x\right)=\{\begin{array}{cc} 1, & \text{if}\;\;x<-\varepsilon ,\\ 0, & \text{if}\;\left|x\right|>\varepsilon ,\\ \frac{1}{2}\left[1+\frac{2}{\pi }\arctan\left(\frac{x}{\varepsilon }\right)\right], & \text{if}\;\left|x\right|\leq \varepsilon . \end{array} \end{array}$$

The values used in the simulations are *ε* = *h* = 1 with *h* denoting the space step.

In each step, the *ϕ*(*x*, *y*) should be reinitialized to be the signed distance function [24, 26]. This procedure prevents the level set function from becoming too “flat” due to the use of the regularized Dirac Delta function *δ*_*ε*_(*x*) [26]. The reinitialization process is expressed as

(19) $$\begin{array}{c} \frac{\partial \psi }{\partial \tau }=\mathrm{sign}\left(\phi \left(x,y,t\right)\right)\left(1-\left|\nabla \psi \right|\right),\\ \psi \left(0\right)=\phi \left(x,y,t\right). \end{array}$$

The solution of this equation *ψ* will have the same zero-level-set as *ϕ*(*x*, *y*, *t*), and |∇*ψ*| will converge to 1 since it should be a distance function.

The process of the vascular extraction terminates when the evolution does not change within bounds 0.4 mm^2^ on successive iterations or the maximum number of iterations is reached. The improved active contour method converges to the boundary of vascular structures exactly in a few iterations.

### 3.3. Implementation of the Algorithm

1. Vessel enhancement with morphological top-hat transformation and Hessian-based multiscale filtering;
2. Get the initial contour *C*_0_ using Otsu's thresholding method on the enhanced image *ν*_*I*_;
3. Initialize *ϕ* ^0^ based on *C*_0_;
4. Compute *c*_1_(*ϕ*) and *c*_2_(*ϕ*);
5. Solve (17);
6. Reinitialize *ϕ* ^*n*+1^ as *ϕ* ^*n*+1^ = 0 by using (19);
7. Check whether the solution is stationary or the stopping criteria is met. If not, go back to Step 4; Otherwise stop evolution.

## 4. Experiments and Discussions

### 4.1. Experiments on Synthetic Images

 To evaluate the performance of the proposed method, we test it on synthetic images containing different vessel-like structures with different diameters and different directions. For quantification, we use the segmentation rate to measure the effectiveness of our method. The segmentation rate is used to estimate the completeness of a segmented vessel, and it is defined as the ratio of the number of segmented pixels to the number of gold standard pixels whose coordinates are known in synthetic images.

#### 4.1.1. Evaluation of Segmentation Accuracy and Robustness

**Figure 1:**
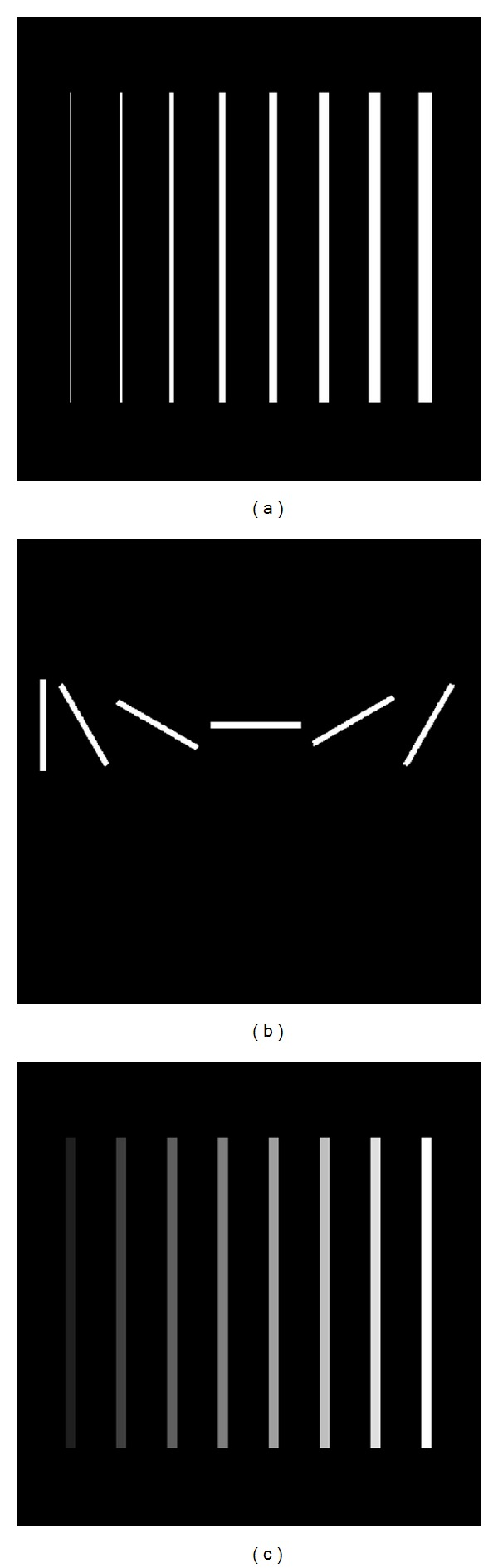
Synthetic images. (a) Image with different diameters. (b) Image with different directions. (c) Image with different intensities.

**Figure 2:**
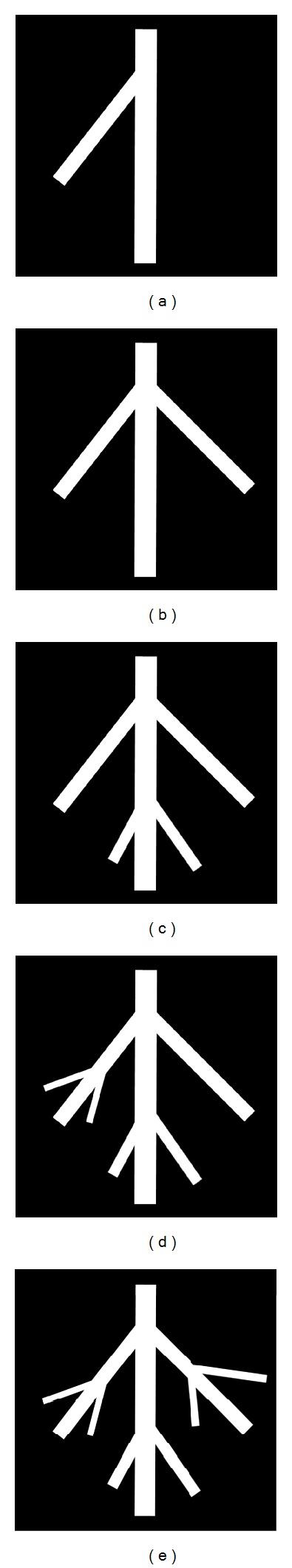
The vascular tree model with different complexities. (a) A branch. (b) Two branches. (c) Four branches. (d) Six branches. (e) Eight branches.

Figure 1 shows three types of synthetic images of size 512 × 512. In Figure 1(a), the diameters of the simulated vascular structures range from 1 pixel to 15 pixels. In Figure 1(b), directions of the vascular structures are simulated by counter-clockwise rotation with an interval of 30 degrees starting from the vertical direction. In Figure 1(c), the intensities of the vessels from left to right are set between 32 to 256 with an increment of 32, while the intensity of background is 0. Meanwhile, five vascular tree models with different complexities are presented in Figure 2.

**Figure 3:**
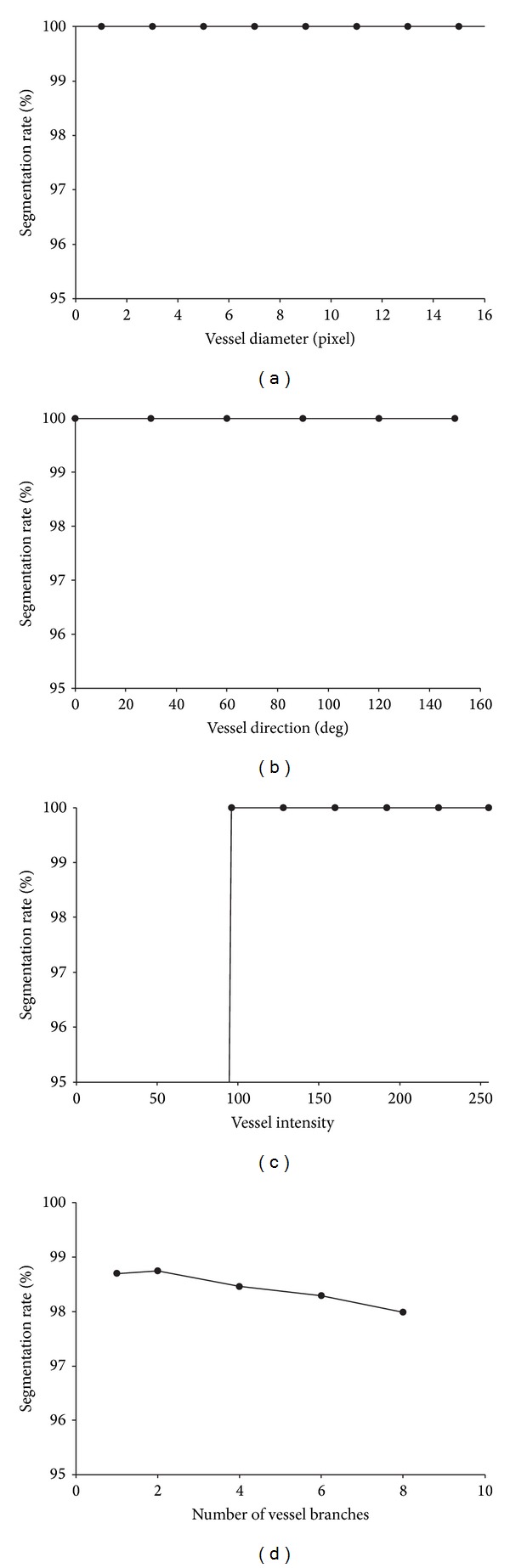
The segmentation rate with different (a) vessel diameters, and (b) vessel directions, (c) vessel intensities, (d) vessel complexities.

Figure 3 shows the segmentation rate with different vessel diameters, vessel directions, vessel intensities, and vessel complexities. It can be seen that the proposed method can provide accurate segmentation results, and the segmentation rates for all the synthetic images are over 95%. Moreover, the segmentation accuracy is insensitive to vessel diameter or vessel direction and all the cases could be segmented completely. Unfortunately, it has a limit on vessel intensity. If a vessel structure is not obvious compared with the background in the image, the proposed method cannot obtain the expected outcome as shown in Figure 3(c). When the intensity of the vessel structures is lower than 64 with the background intensity equal to 0, the segmentation rate is close to 0. But this kind of vessel structure is rare in clinical practice. Simultaneously, with the increasing complexity of vessel structure, the segmentation rate presents a slight downward tendency as shown in Figure 3(d). Therefore, the proposed method is effective for the extraction in images with vessel-like structures.

**Figure 4:**
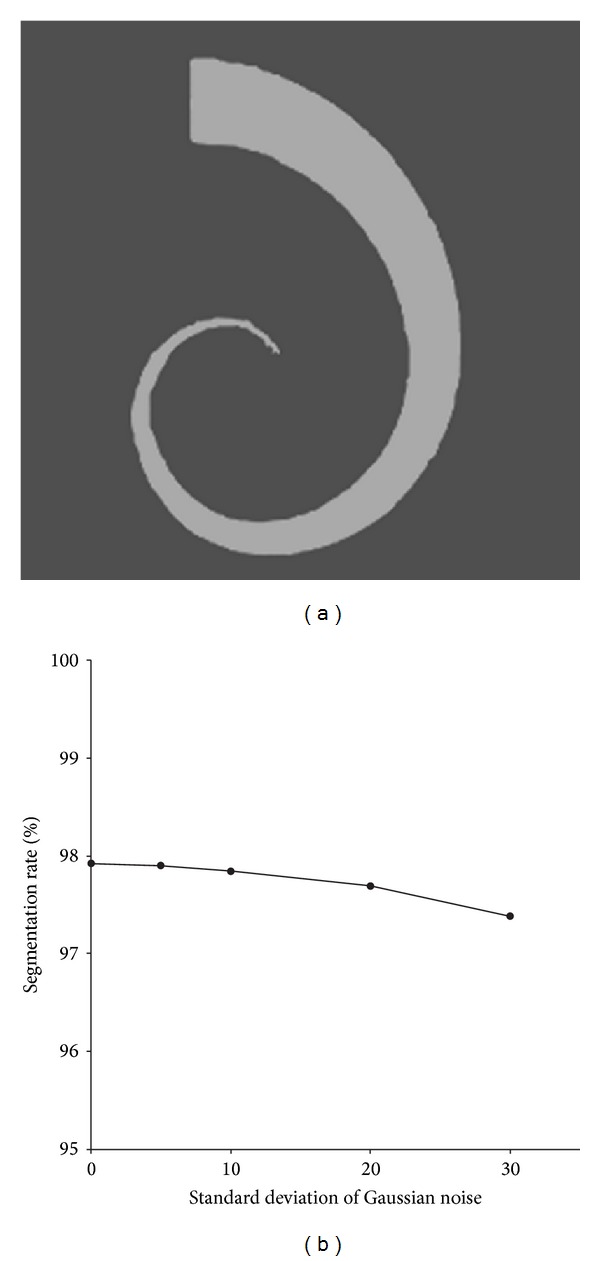
Analysis of noise sensitivity. (a) Original image. (b) Influence of noise levels.

**Figure 5:**
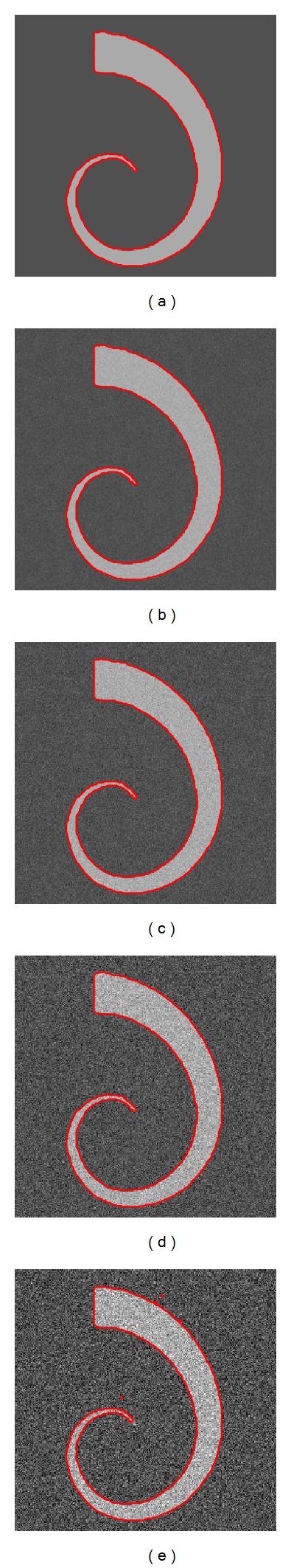
The segmentation results at different noise levels. (a) Original image. Standard deviation is 5, 10, 20, and 30 from (b) to (e).

To investigate the sensitivity of the proposed method to noise, we used the synthetic image of size 256 × 256 with a vessel-like structure of varying width and orientation in Figure 4(a), and added zero mean Gaussian noise of standard deviations ranging from 5 to 30 to this image.  Figure 4(b) shows the segmentation rate for Gaussian noise of the various standard deviations. It is easy to see from Figure 4(b) that the segmentation rate decreases slightly but remains above 97% with increasing noise levels in the image.  Figure 5 shows the segmentation results under different noise levels. Obviously, the proposed method is robust to noise in that it can extract the tree structure effectively at the various noise levels.

#### 4.1.2. Comparison of Vessel Segmentation Methods

**Figure 6:**
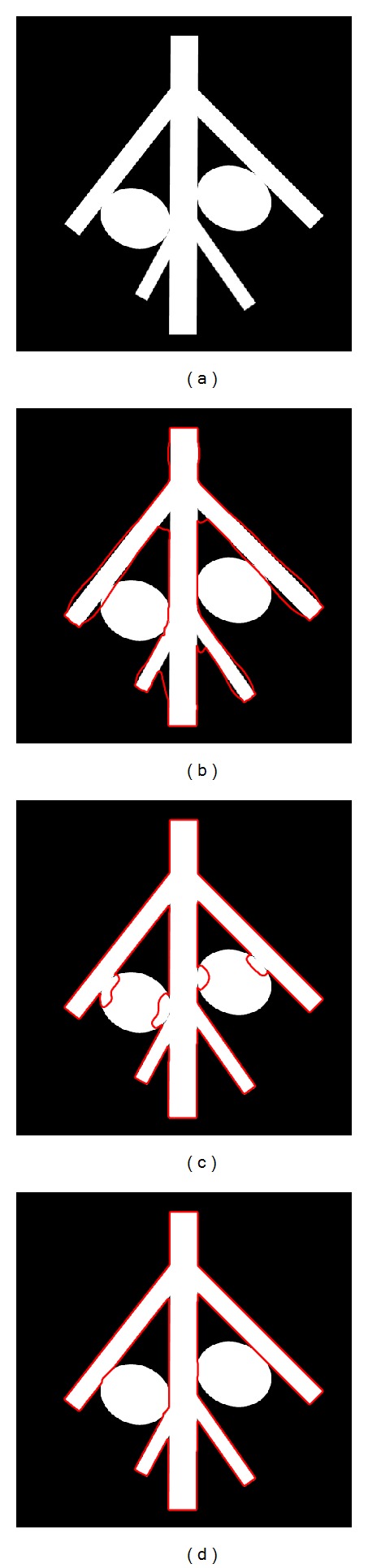
The segmentation results of the above three methods. (a) Original image. (b) Hessian-based multiscale filtering. (c) Hessian-based multiscale filtering combined with Chan-Vese model. (d) The proposed method.

 In this section, we compared the segmentation results of the proposed method with those of other two vessel segmentation techniques, Hessian-based multiscale filtering [5] and Hessian-based multiscale filtering combined with Chan-Vese model [24], on the synthetic image. The synthetic image of size 512 × 512 used in this part is given in Figure 6(a).  Figure 6(b) shows that the Hessian-based multiscale filtering can locate vessel structures accurately but with inaccurate scales, which means that it is suitable to generate the initial contour. Hessian-based multiscale filtering combined with Chan-Vese model can converge to the boundary of the vascular structure exactly, but it will leak into neighboring nonvascular structures where the contrast is low as shown in Figure 6(c). The result of the proposed method presented in Figure 6(d) is of high accuracy and completely unaffected by nonvascular structures because of the introduction of a new class of external constrained term *F*_*σ*_ to penalize the evolution distance of the contour. The segmentation rate of Hessian-based multiscale filtering, Hessian-based multiscale filtering combined with Chan-Vese model, and the proposed method are 88.71%, 92.32%, and 98.14%, respectively.

### 4.2. Experiments on MRA Images

 In this section, we applied the proposed method on 2D slices extracted from a 3D abdomen MRA image. The image size is 512 × 512 × 60 voxels with pixel spacing 0.51 mm × 0.51 mm × 1 mm which is acquired from syngo MR B15 by routine clinical scan. The 2D slices were generated by slicing through the 3D image in the direction of the coronal plane with 3D Quantify (a multiplanar visualization software) [27]. For the clinical images, there is no “ground truth” to prove presence or absence of the vessel structures or their sizes or positions. Thus, we evaluated segmentation results of the proposed method and the other two methods by visual inspection.

**Figure 7:**
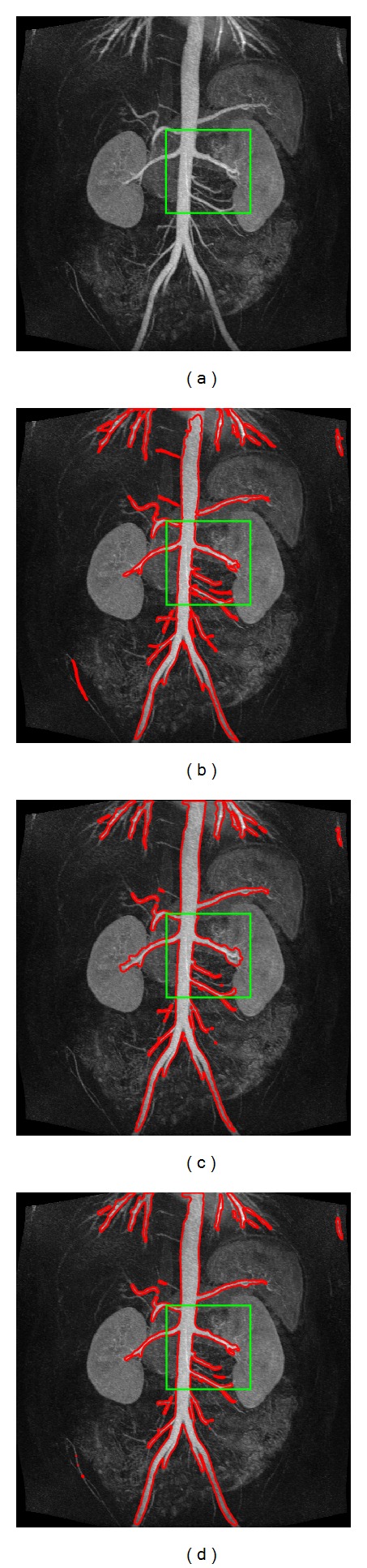
Segmentation results of the above three methods. (a) A 2D slice view. (b) Hessian-based multiscale filtering. (c) Hessian-based multiscale filtering combined with Chan-Vese model. (d) The proposed method.

**Figure 8:**
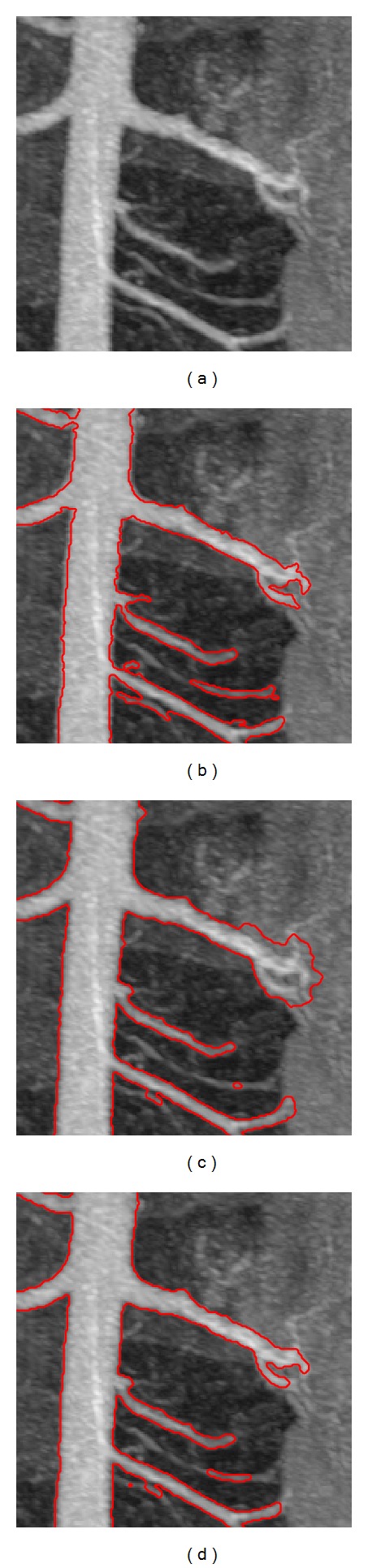
Enlarged view of the marked green box in Figure 7. (a) A 2D slice view. (b) Hessian-based multiscale filtering. (c) Hessian-based multiscale filtering combined with Chan-Vese model. (d) The proposed method.

Figure 7(a) shows one of the 2D slices used in our experiments. We compared our proposed method with the Hessian-based multiscale filtering and Hessian-based multiscale filtering combined with Chan-Vese model. The results are presented in Figures 7(b), 7(c), and 7(d).  Figure 8 shows a partially enlarged view of the marked green box in Figure 7. Obviously, Hessian-based multiscale filtering cannot estimate the scales of the vessel structures exactly inferred from the segmentation of the main renal artery on the left of Figure 8(b). Similar to Figure 6(c), the result of Hessian-based multiscale filtering combined with Chan-Vese model leaks into adjacent nonvascular structures due to the low contrast as shown on the right side of Figure 8(c).  Figure 8(d) demonstrates that the proposed method successfully and accurately extracts most of the vascular structures.

## 5. Conclusions

 This paper presented an automatic technique for extracting vascular tree in medical images. Distinctively, the proposed method introduces an external constrained term *F*_*σ*_ based on *σ* used in Hessian matrix with Gaussian function convolution into the level set to avoid the segmentation leakage of nonvascular structures. In the evaluation based on synthetic images, the segmentation rate of the proposed method is over 97% and it is robust to noise. The results for clinical datasets demonstrate that the proposed method is suitable and effective for the extraction of vascular tree in medical images. The main drawback of our method is that it cannot obtain expected results when the image contrast is very low. Future work will concentrate mainly on optimizing the performance on low contrast images and accuracy evaluation of our method for clinical datasets.

## Figures and Tables

**Table 1:** Possible structure orientations in 3D images depending on the eigenvalues of Hessian matrix.

Orientation pattern	3D image		
	*λ*_1_	*λ*_2_	*λ*_3_
Noisy, no preferred direction	L	L	L
Plate-like structure (bright)	L	L	H−
Plate-like structure (dark)	L	L	H+
Tubular structure (bright)	L	H−	H−
Tubular structure (dark)	L	H+	H+
Blob-like structure (bright)	H−	H−	H−
Blob-like structure (dark)	H+	H+	H+

L: Low, H+: high positive, H−: high negative.
